# Examining provider perceptions and practices for comprehensive geriatric assessment among cancer survivors: a qualitative study with an implementation science focus

**DOI:** 10.3389/fragi.2023.1305922

**Published:** 2023-12-04

**Authors:** Aaron T. Seaman, Julia H. Rowland, Samantha J. Werts, Rowena M. Tam, Tara K. Torres, Freda Allyson Hucek, Karen E. Wickersham, Ciaran M. Fairman, Hiten D. Patel, Cynthia A. Thomson, James R. Hebert, Daniela B. Friedman

**Affiliations:** ^1^ Department of Internal Medicine - General Internal Medicine, Carver College of Medicine, University of Iowa, Iowa City, IA, United States; ^2^ Smith Center for Healing and the Arts, Washington, DC, United States; ^3^ Department of Health Promotion Sciences, Mel and Enid Zuckerman College of Public Health, University of Arizona, Tucson, AZ, United States; ^4^ University of Arizona Cancer Center, Tucson, AZ, United States; ^5^ Physical Therapy Program, School of Exercise and Nutritional Sciences, San Diego State University, San Diego, CA, United States; ^6^ Herbert Wertheim School of Public Health and Human Longevity Science, UC San Diego, San Diego, CA, United States; ^7^ Department of Psychology, College of Science, University of Arizona, Tucson, AZ, United States; ^8^ Office for the Study of Aging, Department of Health Promotion, Education, and Behavior, Arnold School of Public Health, University of South Carolina, Columbia, SC, United States; ^9^ College of Nursing, University of South Carolina, Columbia, SC, United States; ^10^ Department of Exercise Science, Arnold School of Public Health, University of South Carolina, Columbia, SC, United States; ^11^ Department of Urology, Feinberg School of Medicine, Northwestern University, Chicago, IL, United States; ^12^ Department of Epidemiology and Biostatistics, Arnold School of Public Health, University of South Carolina, Columbia, SC, United States; ^13^ Cancer Prevention and Control Program, Arnold School of Public Health, University of South Carolina, Columbia, SC, United States

**Keywords:** implementation science, geriatric oncology, cancer survivorship, qualitative research, aging

## Abstract

**Introduction:** Cancer rates increase with age, and older cancer survivors have unique medical care needs, making assessment of health status and identification of appropriate supportive resources key to delivery of optimal cancer care. Comprehensive geriatric assessments (CGAs) help determine an older person’s functional capabilities as cancer care providers plan treatment and follow-up care*.* Despite its proven utility, research on implementation of CGA is lacking.

**Methods:** Guided by a qualitative description approach and through interviews with primary care providers and oncologists, our goal was to better understand barriers and facilitators of CGA use and identify training and support needs for implementation. Participants were identified through Cancer Prevention and Control Research Network partner listservs and a national cancer and aging organization. Potential interviewees, contacted via email, were provided with a description of the study purpose. Eight semi-structured interviews were conducted via Zoom, recorded, and transcribed *verbatim* by a professional transcription service. The interview guide explored providers’ knowledge and use of CGAs. For codebook development, three representative transcripts were independently reviewed and coded by four team members. The interpretive process involved reflecting, transcribing, coding, and searching for and identifying themes.

**Results:** Providers shared that, while it would be ideal to administer CGAs with all new patients, they were not always able to do this. Instead, they used brief screening tools or portions of CGAs, or both. There was variability in how CGA domains were assessed; however, all considered CGAs useful and they communicated with patients about their benefits. Identified facilitators of implementation included having clinic champions, an interdisciplinary care team to assist with implementation and referrals for intervention, and institutional resources and buy-in. Barriers noted included limited staff capacity and competing demands on time, provider inexperience, and misaligned institutional priorities.

**Discussion:** Findings can guide solutions for improving the broader and more systematic use of CGAs in the care of older cancer patients. Uptake of processes like CGA to better identify those at risk of poor outcomes and intervening early to modify treatments are critical to maximize the health of the growing population of older cancer survivors living through and beyond their disease.

## 1 Introduction

Since 2018, the gerontologic community and the American Society of Clinical Oncology (Mohile et al., 2018) have recommended use of comprehensive geriatric assessments (CGAs) in the care of older adults diagnosed with cancer. However, there is great variability in the extent to which the CGAs are currently used. This study involved discussions with primary and oncology care providers to better understand their use of these assessments among patients with cancer.

The Cancer Prevention and Control Research Network (CPCRN) is a Centers for Disease Control and Prevention (CDC)-funded collaborative. The current CPCRN network consists of a funded coordinating center and eight funded research institutions (Leeman et al., 2019; White et al., 2019; Wheeler et al., 2023). The network has extensive experience conducting cancer control interventions of various types and in partnering with communities and clinical partners in dissemination and implementation (D&I) research and evaluation activities (Wheeler et al., 2023). Many of these efforts focus either directly on patients with cancer or individuals at high risk of cancer and other chronic disease comorbidities. The CPCRN’s Cancer Survivorship Workgroup consists of over 70 members across all funded CPCRN centers and affiliate members. Its overall goal is to advance interdisciplinary research collaborations that support cancer survivorship science and outreach to promote health equity among cancer survivors. Workgroup members define a cancer survivor and survivorship as follows: “An individual is considered a cancer survivor from the time of diagnosis, through the balance of her/his/their life. The term also includes secondary survivors, such as caregivers and family members of those diagnosed with cancer who are also affected by the cancer journey” (Cancer Prevention and Control Research Network, 2023). Survivorship encompasses the entire lived experience of survivors, including health-related quality of life (Rowland and Bellizzi, 2008; Rowland and Bellizzi, 2014). An older cancer survivor is generally defined as an individual diagnosed with cancer after age 65 years.

Cohen’s seminal 2007 article on the interface of cancer and aging provides recommendations for research in three main areas: basic biology, societal and psychosocial aspects, and clinical aspects (Cohen, 2007). These broad, potentially intersecting, categories include multiple focus areas such as cancer screening and survivorship, barriers to participation in clinical trials and design of trials for older adults, cultural differences, family communication, tumor behavior, inflammation, comorbidities, and physical and cognition function. Survivorship science encompasses the physical, psychological, social, economic, and spiritual health of survivors across the cancer continuum, with special emphasis on long-term wellbeing, including disease prevention and health promotion over the life course. Older cancer survivors have unique survivorship needs, including multiple chronic conditions (Parekh and Goodman, 2013), decreased functional status, polypharmacy, and risk for social isolation and financial toxicity; therefore, having relevant, comprehensive assessment of their needs, and information and resources designed to meet these needs is critically important (Rowland and Bellizzi, 2008; Rowland and Bellizzi, 2014; Doi et al., 2023).

CGAs provide a means for identifying these needs early so that appropriate intervention and care planning can occur (Culakova et al., 2023; Doi et al., 2023; Lin et al., 2023; Singhal et al., 2023). CGAs are “multidimensional, interdisciplinary diagnostic process[es] focusing on determining an older person’s medical, psychosocial, and functional capabilities to develop a coordinated and integrated plan for treatment and long-term follow-up” (Ellis et al., 2017; Hamaker et al., 2022; Outlaw et al., 2022). While much work has been done to develop recommendations for CGAs using validated instruments (Mohile et al., 2018; Hamaker et al., 2022; Dale et al., 2023), research on the actual implementation of CGA processes within primary and specialty care settings for vulnerable populations of aging cancer survivors is lagging. Research is critically needed to identify the barriers to inclusive, systematic assessment and potential solutions to address those barriers. The purpose of this research is to better understand the barriers and facilitators of geriatric assessment use and increase awareness about these types of assessment tools. The long-term goals of this work are to: a) provide recommendations for feasible and pragmatic implementation of a CGA, b) to suggest next steps or pathways for follow-up based on CGA results among older cancer survivors, and c) to increase the capability of clinic staff to critically engage with and implement CGAs among older cancer survivors.

## 2 Methods

English-speaking primary care and cancer care providers were identified through CPCRN partner listservs across the United States and with the support of a national cancer and aging research organization. Information shared involved a description of the study purpose and contact information for one of the authors (DBF) who conducted the interviews. Participants were informed that these discussions are intended to help plan strategies, trainings, and the creation of resources for broader implementation of CGAs in primary care and oncology care settings, as well as to identify barriers to implementation.

Semi-structured interviews lasted between 30–60 min and were conducted by one author (DBF) via Zoom (Zoom, 2022), recorded, and transcribed *verbatim* by Ubiqus, a professional transcription service. The interview guide (see Appendix 1), pilot-tested with two partner providers in advance, had 21 questions and explored providers’ current knowledge and use of geriatric assessments in their clinics. Interview questions focused on providers’ perceived purpose and value of CGAs; benefits and barriers to administering a CGA; details about implementation, including measures currently being employed and how results are used; and ideas for trainings that might improve implementation.

Domains specified by the International Society of Geriatric Oncology (International Society of Geriatric Oncology SIOG, 2023) were reviewed with participants to see if they implemented some or all of the following measures: functional status check (e.g., ADLs/IADLs), cognitive function assessment, Geriatric Depression Scale, nutritional assessment (e.g., Mini Nutritional Assessment/MNA), gait and balance assessment (e.g., Timed Get Up and Go/TGUG), Cumulative Illness Rating Scale for Geriatrics, Comorbidity Index (e.g., ACE 27), quality of life assessment (e.g., QLQ-C30).

Original interview audio files were securely stored in a protected cloud-based storage application with limited team member access. Transcribed interviews were de-identified (Participant 1, Participant 2, etc.). A qualitative description design guided the coding and analysis approach. Such a design is often used for healthcare research with providers and/or within a healthcare setting where the topic is salient and data is collected despite time and resource limitations (Bradshaw et al., 2017). Using a deductive analytic approach, four team members (SJW, RMT, TKT, DBF) used the interview guide to develop the list of categories for initial open coding. Using an inductive process, team members then independently reviewed and coded three of the eight transcripts to flesh out and finalize the codebook. In-depth discussions among all coders revealed complete agreement on the coding for these two initial transcripts. Four team members (SJW, RMT, TKT, and FAH) proceeded to fully code manually all eight transcripts (two transcripts each) for theme development and analysis (Vaismoradi et al., 2016). This process involved reflecting, transcribing, coding, and searching for and identifying themes and another group discussion following individual coding to ensure complete agreement (Braun and Clarke, 2014; Braun and Clarke, 2023). Participant responses are presented according to the following four overarching thematic categories:

• Perceived purpose and value of CGAs and how the need for CGAs is communicated with cancer patients
• Current implementation practices
• Perceived barriers and facilitators to implementing CGAs
• Participants’ training preferences and needs

We employed a rigorous and systematic approach to our qualitative methods and analysis. Our work aligns with the Standards for Reporting Qualitative Research (SRQR) as presented in Supplementary Appendix SA2 (O Brien et al., 2014). All human subjects related protocols were approved by the University of South Carolina Office of Research Compliance.

## 3 Results

### 3.1 Summary of provider demographics

Eight Zoom-based interviews with nine providers across the United States (two individuals participated in one interview) were conducted between December 2022 and February 2023. Of the nine providers, eight worked in specialty care (geriatric oncology, genitourinary oncology, hematology) and one was a primary care/internal medicine provider. Regarding their affiliations, six participants were affiliated with academic medical centers/cancer centers, one was connected with a VA hospital, and two were in independent practices.

Table 1 presents the domains with relevant measures that providers were assessing with cancer patients. Five participants indicated they conducted partial CGA assessments with their patients and identified the tools they used to assess each domain. Only one provider systematically assessed all eight recommended domains. Two individuals indicated they were not implementing components of a CGA; however, they were able to engage with all other interview questions. Given that this was a small sample and that individuals in the field focused on CGAs are associated with the same national organizations, we did not collect additional demographic details from interview participants.

**TABLE 1:** CGA domains being used by primary and oncology care providers interviewed (Source: SIOG) (N = 8).

CGA domains	# Providers assessing each domain	Assessment tools being used
Functional status	5	IDLs/IADLs (Wheeler et al., 2023); Observation (Mohile et al., 2018); SF36 (Mohile et al., 2018)
Cognition	6	Mini-Cog (Leeman et al., 2019); MMSE (Mohile et al., 2018); Mini-Cog/MOCA (Mohile et al., 2018); PROMIS (Mohile et al., 2018); Blessed Test, then MOCA (Mohile et al., 2018)
Depression	6	Geriatric Depression Scale (Wheeler et al., 2023); PROMIS (Mohile et al., 2018); GDS as part of SAOP (Mohile et al., 2018); Screening questionnaire (Mohile et al., 2018)
Nutrition	5	Mini Nutrition Assessment (Wheeler et al., 2023); BMI calculation (Mohile et al., 2018); Patient Generated Subject Global Assessment (Mohile et al., 2018)
Gait and balance	6	Clinic question (Mohile et al., 2018); Performance battery (Mohile et al., 2018); Short Physical Performance Battery and grip strength (Mohile et al., 2018); Short Physical Performance Battery (Mohile et al., 2018); Observation (Mohile et al., 2018); Fall history (Mohile et al., 2018)
Cumulative illness rating	2	Considered part of comorbidity (Mohile et al., 2018); “specific calculator” (Mohile et al., 2018)
Comorbidities	6	Geriatric-specific Comorbidity Index (Wheeler et al., 2023); Charleston Comorbidity Index (Leeman et al., 2019); Chart review (Mohile et al., 2018)
Quality of life	4	Considered advanced care planning discussion (Mohile et al., 2018); PROMIS Global-10 (Mohile et al., 2018); SAOP3 (Mohile et al., 2018); Self-reported quality of life (Mohile et al., 2018)

### 3.2 Presentation of qualitative themes

Qualitative interview findings are presented according to the four thematic content categories identified (Mohile et al., 2018): purpose and value of a CGA and how this is communicated to patients and families (Leeman et al., 2019), CGA administration logistics (Wheeler et al., 2023), barriers and facilitators to CGA implementation, and (White et al., 2019) implementation training preferences and needs.

#### 3.2.1 Purpose and value of a comprehensive geriatric assessment with cancer patients

Participants shared that the main purpose of conducting a CGA with patients was to assist with clinical decision-making, treatment planning, and to facilitate care coordination.

Regarding decision-making and treatment planning, participants noted that implementing a CGA allowed them to better understand the patient’s overall health status. These data then serve to guide any needed intervention or changes in treatment plans as well as help tailor cancer treatments to address patients’ needs.

*We do the assessment, plus we intervene for decision-making. So the first value is the decision-making piece. Understanding the health status will help us guide cancer treatment plans.* Participant 2

*I think the idea is, you know, better assessing the older adult to inform treatment decision making and guide treatment plans, um, and really the, the personalization of their cancer treatment to avoid overtreatment and undertreatment but to, to do the right treatment.* Participant 4

*Whether they bring up the matter or not I tell them that it is important for us to do, um, an evaluation. A more comprehensive evaluation that will be able to help both the patient and I. To make a decision about what treatment they might be able to tolerate.* Participant 3

Care coordination was another important purpose for conducting a CGA. Having the entire picture of a patient’s health through a CGA helped guide the coordination of care and involve appropriate care team members. Participants shared they were better able to understand what was needed for their patients to provide the best care in terms of a team of healthcare professionals.

*Here’s how we coordinate their care with cardiology and all of this stuff. So that’s more of internal medicine… [and] geriatrics.* Participant 2

*I think that doing geriatric assessment has great value because for me if I want to put a word for that, it’s like you are doing 360 evaluation for your patient. You are not just focused on, you know, a specific disease, specific comorbidity and that—sometimes we are or other providers that were not doing the geriatric assessment or geriatric assessment types of tests. Um, we are missing the whole picture. That’s why I talk with my people.* Participant 6

All providers expressed the value of implementing CGAs with cancer patients. Although not all providers were implementing full CGAs due to various barriers (presented in a separate section of the Results), providers unanimously agreed there is value in implementing CGAs. One of the specific values of CGA implementation highlighted by providers is its use as an initial screening tool for patients.

*I think that the benefit in the screening tools is in standardizing that feeling that we all have…I feel like there’s a lot of bias—implicit bias—in how we, as physicians, think about giving chemotherapy to the elderly, and that it is not one size fits all, you know? Different people at different ages have different capacities. So, I like the screening because it standardizes things.* Participant 8

*So a geriatric assessment which is a multidimensional evaluation of a number of domains that a regular oncologist normally will not evaluate, that assessment allows you to… uncover problems that normally you will not uncover. Because it gives you a multidimensional assessment of an older person with cancer who normally you will not get with the standard oncology tools. Um, an older individual with cancer, uh, is a complex human being. Certainly, because with aging there’s changes in physiology, um, and changes in their entire body. And the treatment of cancer can have certain detrimental effects on such an older individual that you might not experience with a younger individual.* Participant 3

Another value providers discussed was the added support it provided for patients. This ranged from facilitating discussions with patients about survivorship to the overall level of clinic support the patient received.

*And I suppose the difference in when you’re in a cancer survivorship kind of mindset is, you know, are they having—if they’re going through treatment are they having any particular side effects or problems with this treatment. Does their treatment plan need to be adjusted so yeah. I, I do think. I do not know that if that’s a function of the geriatric assessment by itself or if it’s, you know, you have to be in a mindset of really thinking about survivorship.* Participant 1

*Because I do feel like I head things off [by doing a CGA]. I take better care of patients and put supports in place that make their experience better.* Participant 5

Most providers indicated that they provided their patients with a high-level overview of the purpose of the CGA prior to administration. A few participants shared that they explain the concept of the heterogeneity of aging to describe why geriatric assessments are necessary for making care decisions.

*I’ll usually say something like you know, you may know people who are the same age as you but they have very different medical issues… I’ll say something like, you know, you may know people who are your age but they have memory issues and they live in a nursing home or, you know, other things. So you know, the age does not really tell us very much about you and it’s our job to really get to know you on all these levels.* Participant 5

To explain why CGAs were conducted in an oncology clinic, many providers indicated that they discussed with patients how assessments informed cancer care decisions and how any age-related concerns might relate to treatment tolerance.

*These older adults are very… in tune with their body. So one of the first questions they’re going to ask you is do you think I can tolerate treatment. So it makes the conversation very easy. Whether they bring up the matter or not I tell them that it is important for us to do… an evaluation. A more comprehensive evaluation that will be able to help both the patient and I to make a decision about what treatment they might be able to tolerate.* Participant 3

The overall approach of providers was to describe how CGAs provided extra support for both the patient and the provider when making care decisions. CGAs allowed for personalized and tailored treatment recommendations and potentially needed interventions prior to the start of any cancer treatment place that would be put into place. One provider indicated that they provided education to their patients about their risk status during and after conducting the CGA.

*I go through it. I’m very like ok, here’s the domain. Here’s the problem and here’s the, you know, clinical process outcome. So I look at the scores for each domain and I talk to the patients about that. And then for each domain as I’m like talking to the patient I’m running it down in my head. Ok. Functionally here’s what’s going on. You’re falling. Here’s what we should do.* Participant 2

#### 3.2.2 Logistics of administering a CGA

When asked about the logistics and protocols for administering a CGA, participants discussed the time it took to implement assessments and the need for multidisciplinary team coordination to do so. Administration, documentation, and follow-up referrals to care were quite variable.

##### 3.2.2.1 Administration time

Participants indicated that the assessment generally took between 30 minutes to an hour to complete in their clinic, but the time required was largely variable, depending on patient needs.

*I would say… it’s variable. Like the patient I had today took a long time ‘cause she had… moderate dementia. I mean just like explaining the tests to her, getting through a MoCA… it takes a long time. Other patients… they fill out the survey in 10 min.* Participant 5

##### 3.2.2.2 Multidisciplinary team involvement

For the clinics implementing CGAs, most indicated that they had a multidisciplinary team who completed assessments for each domain. This team may have included the oncologist, physical therapist, occupational therapist, pharmacist, nurse navigator, or other advanced practice provider. They expressed that this model helped ease the burden on them, and it was suggested that appointing specific medical staff who specialized in geriatrics, nurse navigators, social workers, and other specialists helped with effective implementation.

One geriatric oncology provider indicated that they had a clinical coordinator who managed the clinic flow for new patients.

*She… coordinates in the clinic who is going to see the patient. And who is in the room. So she makes sure the patients are just not sitting there by themselves for a long time… She kind of starts the process by making sure the assessment is completed.* Participant 2

When asked if all care center staff were on board with implementation of geriatric assessments, responses were mixed. Most indicated that, in general, care center staff were on board; however, not all staff recognized the value or importance of the assessment.

*It is very tough. I know certain centers, a few centers have done well and have a dedicated geriatric oncology clinic. Some call it senior adult oncology program. There’s a wide variety of them. We do not have that at [center name]* … *So* … *when I do the consultation* … *I have to refer them for any and all interventions.* Participant 3

##### 3.2.2.3 Variable administration, documentation, and referrals

Even within clinics that administered components of CGAs, there was not always a systematic approach for how and when the assessment was conducted. Most commonly, participants indicated that the CGAs were administered as part of the intake process for a new patient.

*We’ve now embedded this in the typical intake process for solid tumor oncology in [medical oncology]. So when… I have a new patient appointment to see medical oncology, you know, that’s really who gets them the most.* Participant 4

Because the assessment was often time-consuming and clinic staff capacity may be low, some participants indicated that they scheduled follow-up visits for patients who may have greater geriatric-related needs.

*I normally am not able to do that assessment on that same day that I’m doing my initial consultation. It by itself is a full 1 h where you have to go through the diagnosis treatment options. So I usually will schedule them for a separate visit to come in to complete that geriatric assessment.* Participant 3

Some clinics provided patients with the assessment questions to complete ahead of their visit, while other providers incorporated CGA questions during the visit when asking patients about their medical history.

*I lean towards a number of the COG instruments. Simply because most of them are patient self-administered. And it works very well because when I see you I can give you a packet of questions to take home with you ahead of the visit the following week for the geriatric assessment*. Participant 3

*What I do is I basically just fill in the G8 or SAOP3 in my questioning of their history. So, I do not make them fill it out. We do not have iPads or a screening thing so, I will ask them like, specific questions in their history that will basically tell me about their G8 and the numbers, ‘cause it is enough to do the scoring.* Participant 7

When determining which patients would receive a CGA, two clinics used a threshold of 65 or 70 years of age. All patients meeting the criteria received the assessment. Other clinics used a shorter initial screening (i.e., not the full CGA) to identify which patients were at higher risk to allocate limited resources better.

*The screening is a very simple two, 3-min questionnaire. Very simple and then most people actually do not need a geriatric assessment. And if you would need a geriatric assessment they get the geriatric assessment.* Participant 3

Most clinics documented the CGA results in the electronic medical record (EMR), either in the summary notes for the visit or in a dedicated template. One participant indicated that within the template was information about the assessment to help facilitate scoring and interpretation.

*We have integrated it into… the electronic medical record and have essentially made dashboards that try to distill this information down to help those that, you know, do not understand the field per se.* Participant 4

Follow-up and referral to other care prior to cancer treatment was based on both the patients’ identified geriatric needs and their oncology needs. In many cases, a determination for the next steps and recommendations for care were based on balancing risks and benefits. One provider gave the following example of balancing geriatric needs with oncology needs while talking with a patient and their caregiver:

*They might have one medical problem that bothers them. And then you’re like, you know, yes, treatment is in your best interest ‘cause the cancer is aggressive… you need to do the chemo. If she exercises and she does all these other things, accelerated aging we can work on. But if you do not do the chemotherapy—Your mom might not be a survivor.* Participant 2

#### 3.2.3 Barriers and facilitators to implementation of CGAs in cancer care

Participants shared several barriers and facilitators at multiple levels of the healthcare system related to the implementation of CGAs in cancer care. Notable barriers included time, lack of a multidisciplinary team, limited knowledge about the purpose of CGAs and training in implementation, limited championing at the organizational or system level, limited incentives regarding billing, and family members’ competing priorities. Facilitators to implementation were having top-level institutional buy-in, provider education and training, multidisciplinary teams in place, and clearly communicating about CGAs with family members and caregivers.

##### 3.2.3.1 Barriers to implementation

###### 3.2.3.1.1 Limited time

Most participants noted that the issues of time allotted for each patient encounter and the time needed to conduct the assessment did not contribute to a conducive environment for regularly implementing the assessment. One participant reported needing to accommodate other assessments or discussions with the patient during their appointment to ensure they covered other topics that were pertinent to their role as an oncologist.

*I think it is the practitioner experience level and administration of the test and how to either—is it best to have someone ask these things over the phone, have the patient sit and do it his or her self, and then, just really having—that first point of contact is really important, because it is—I think, for physicians, it is not—especially for cancer physicians—we have so much other stuff to discuss, it is tough to do—regularly, too, you know?* Participant 7

The issue of time was identified as particularly problematic when participants considered the difference in length of time that they typically needed between their older- and younger-aged patients. One participant described their observations of different aged patients and the need to give more time to older patients to allow for more questions and discussion from the patient. The unique age-related accommodations that were needed during oncology appointments further infringed on the time needed to administer CGAs.

*The first just being time. Like, today, the way things are currently set up, my barrier is time—especially because it is this patient population that I end up feeling like I spend more time with to begin with. My younger patients, I can kind of explain things quickly, move through treatment options. It is this patient population that requires, in my experience, more explanation, more information. And then, you’re adding on top of it this whole screening tool.* Participant 8

###### 3.2.3.1.2 Lack of multidisciplinary team administration and collaboration

Participants shared that not having a multidisciplinary team behind them to help with the administration and management of the CGAs was another significant barrier to implementation. Participants discussed the need for their institution to have teams who specialized in geriatrics and could provide more expertise in the roll-out of administrating these assessments. Relatedly, participants also discussed that their institution tended to be more siloed in nature, and they did not get the opportunity to work in integrated teams. The lack of team administration and planning could preclude providers from ensuring that concerns arising from the CGAs are routinely monitored, prioritized, and managed across multiple providers on a patient’s oncology medical team.

*I think the—I think in the clinic, I think in primary care we really need to take that team approach. I get the sense that in cancer settings it’s not just having the provider that has that specific expertise. It is the people working with the provider. They know how to fill out these forms. They know how to, you know, where the resources are going to be because that’s what they do day in and day out.* Participant 1

*If I wanted to take that on, it would be something I would be doing myself in clinic, which is why, a lot of the time, it does not happen.* Participant 8

*So, it is challenging, ‘cause we do not have a true multidisciplinary clinic like some of my colleagues do—specifically for older adults—with all of those people together. Like, we have with our radiation oncologists and surgeons, where I work with them in clinic, but we’re all physicians and we do not have that team—that extra team that we need.* Participant 7

###### 3.2.3.1.3 Limited provider knowledge about the value of CGAs and training on CGA implementation

Most participants identified having a lack of training or experience with implementing aspects of the CGA and interpreting results for next steps and/or referrals. In addition, participants expressed concerns about the type of training a provider should have in order to be a qualified administrator of these assessments.

*One thing that we struggle with is what is the training of the person that you’re hiring for that role? Like, as an example, the person who helps me order all my genetic testing and gets me all of my outside records and everything—she does not have any medical training at all. And I get how that decision was made, but it makes it really hard for her to know what she’s supposed to be doing. Or maybe even a better example is like, our clinical trial coordinators. They are supposed to be going through our charts, figuring out what trials people are appropriate for, but they have no medical background at all.* Participant 8

Participants expressed a lack of clarity on the recommendations for best practices of CGAs. Furthermore, participants indicated there should be a standardized protocol for administering CGAs and detailing the use of CGAs from administration to possible intervention prior to treatment. However, some participants did not necessarily have access to recommendations and/or training that further details the steps for conducting CGAs.

*The other thing is, um, the just the understanding like how to use the information.* Participant 2

*There has been conversation at my institution about either hiring somebody who specializes in geriatric oncology or in consulting with groups who do that well, but yeah, there is no standardized screening right now.* Participant 8

Some providers expressed there was limited clarity and transparency regarding which CGAs have garnered empirical support. While providers acknowledge that the evidence around doing CGAs is robust, there remained limited communication about the evidence base and the purpose of administering and using the results of the CGAs to further inform treatment for cancer-specific symptoms and cancer-related quality of life.

*One person cannot do it alone. You need this assessment to be widely acceptable within the oncology community. You need—Scalable instruments that everybody can administer. With their offices. And all of these instruments can be used in that way.* Participant 3

Furthermore, participants described that limited knowledge about CGAs could contribute to a general undervaluing of CGAs amongst oncology care providers. As described by some participants, with a lack of knowledge about the benefit and purpose of CGAs in providing comprehensive cancer care for geriatric patients, this may undermine the importance for implementing CGAs as a standard practice.

*Also some of them that they do not believe it or just like all this extra work with no clear benefit. Yes, to ASCO and SIOG and other. But the physicians they try to spread the word but I think we are making progress*. Participant 6

*I think that is one of the key reasons why perhaps the integration of geriatrics into oncology has not advanced as fast as we would want it. There is despite all the research—Data. And evidence that’s currently available uptake is suboptimal. Grossly suboptimum. A lot of regular oncologists would tell you oh I know how to treat ______. Why do I need a geriatric assessment? So there’s so much work to do in order to educate, in order to, um, improve uptake. And that’s an area of research that I think that resources should be devoted to.* Participant 3

###### 3.2.3.1.4 Limited champions for CGA use at the organizational/system level

Half of the participants identified prominent barriers at the organizational and system levels. While training and experience were cited as important contributors to providers’ use of CGAs in routine practice, there also is a perceived need for policies set by the organization to regulate the administration of CGAs. Participants identified the need for top-level influences that mandated standardized practice of CGAs in cancer care as well as support from providers’ organizations, national accrediting organizations, and the government. As the value of CGAs is championed at the organization level and nationwide, this may have an influence on providers’ own value of these assessments.

*Organization policy to be changed. And I notice like, you know, for example, the institution I used to work and when I come to [institution name] like there is a policy to implement that. So the staff are trained. You know, they all know a thing. And even like working hard to train does not know that.* Participant 6

*I think the only thing I would say is that trying to make it important has to come from the top level to go down, because of just how we think about disparities, research, and things like that.* Participant 7

###### 3.2.3.1.5 Limited incentives via billing

A few participants identified the inability to bill for CGAs as another barrier to the implementation. Participants described this lack of incentivizing CGA administration such that there were no current mechanisms to bill for this assessment and/or there was a lack of communication from the organization level regarding billing. By recognizing CGAs as a billable service and creating the means to bill, the value of doing CGAs may increase, and providers might have more incentive to prioritize these assessments in their encounters with patients.

*The other thing that would facilitate—because at the end of the day if you are spending time doing this it’s an expectation that you should be able to bill for it. So a way in which that would be much easier to do. So that’s been one of the challenges. How do you bill for these services?* Participant 3

###### 3.2.3.1.6 Other priorities of family members and caregivers of the patient

One participant identified concerns from the patients’ caregivers and family members as a potential barrier to focusing part of the appointment on CGAs. In particular, this arose when family members asked why the provider was assessing cognitive needs during an initial oncology appointment. Family members were concerned with and wanted to focus on the patient’s cancer treatment needs. The participants’ strategies to address this with family included describing the purpose of the CGA and explaining the connections between the patients’ neurocognitive functioning and their cancer-related quality of life.

*So, I think, with—like you mentioned neuro-psychological problems and polypharmacy are really important, because it also goes along if they’re having pretty advanced dementia… And start moving conversations more towards palliative care on what their other mental health states are. ‘Cause I’ve had some of those where like, I’ve had patients with advanced Parkinson’s and really advanced dementia, and they had prostate cancer, and I’m like, “Well, this is not a conversation that’s gonna be better for your health. Why are not we focusing on your neuro-psych problem, you know what I mean?” But families do not realize that ‘cause they’re like, “You have cancer. It has to be taken care of.”* Participant 8

##### 3.2.3.2 Facilitators to implementation

###### 3.2.3.2.1 Top-level champion/buy-in

To combat the several barriers to CGA implementation, many participants emphasized the importance of identifying a champion at multiple levels of their healthcare system in order to create buy-in for implementing CGAs within their facility. Identifying oncology providers or individuals in leadership positions at the organization was a pivotal step to incorporating CGAs into standard cancer care practice. Eliciting buy-in at these levels may provide a starting point for training other providers, forming interdisciplinary teams focused on this work, communicating the value of conducting these assessments, and developing organization policies and billing capabilities.

*Leadership. Leadership buy in. That is imperative. Um, programs that have been able to establish. Geriatric oncology programs within their cancer center have huge leadership buy in. If you do not have leadership buy in you’re not going to be able to do this.* Participant 3

*I think our cancer center director is very interested in this topic and growing the attention, maybe, is how I should put it—being paid to geriatric screening. So, if you asked me independently, “Who do you think the champion is?,” it is our cancer center director. However, I do think medical oncologists—or at least the ones I practice with and see on a day-to-day basis—the interest is growing…Big groups—whether it is ASCO or cooperative groups or whatever—including it in their goals for how practice should change has been a huge thing, at least in my experience, in increasing awareness.* Participant 8

###### 3.2.3.2.2 Provider education and training on CGA implementation

Participants identified the importance of having access to educational opportunities and other current team members who could provide training on CGAs. By having a person to train providers in this work, supplemental education on evidence-based instruments being used as part of CGAs could also be provided and further address the barrier of lack of education on standardized protocols for using CGAs in cancer care.

*Um, but when she was the clinic nurse on our team I kind of really spent a lot of time teaching her about the CARG score.* Participant 5

###### 3.2.3.2.3 Multidisciplinary teams in place

Another worthwhile effort that has helped some of the participants in implementing CGAs within their practice involved identifying other clinicians who may continue to monitor and address the patient’s geriatric needs through interventions within their subspecialty. Identifying members at their institution who were invested in addressing the geriatric needs of the patient may allow for regular use of the CGAs among various providers and ongoing surveillance of patients’ geriatric needs throughout the cancer care continuum.

*I think, you know, from our method that’s the best. Now some other methods if you have to do the cognitive screening and some objective measures then, you know, I think that’s going to be harder. You might be able to do like navigators, maybe lay navigators. You know, I do think that they are, you know, could be really involved in this. Or it might be your medical assistants in the clinic. That would help proctor those pieces.* Participant 4

###### 3.2.3.2.4 Effective communication about CGAs with family members

Participants found that explaining the purpose for doing CGAs to caregivers and family members, in addition to the patient, had been a helpful strategy for including these assessments in their work. One participant acknowledged that these conversations did not need to be time-consuming to be effective, but were extremely important as they allowed both the patient and their loved ones to feel comfortable during the appointment. Another patient reported that patients would typically bring another person with them for appointments; thus, this provided an additional opportunity to explain the purpose and value of the CGAs.

*Yes. So that’s a very good question because the very patient who comes in with cognitive impairment is the very patient who needs a geriatric assessment. The good thing is that most patients are asked to come in with family members. And a patient with cognitive impairment will usually come in with somebody called POA. Yes. And so, um, there’s always a family member for us to be able to explain to them for them to understand and to have their loved one go through the assessment, yes.* Participant 3

#### 3.2.4 Implementation training preferences and needs

Most participants expressed that implementation of CGAs would benefit from training and resources. However, they noted that this would need to be tailored to the individual administering it.

*I think the training has to be kind of tailored to whoever your audience really is. You know, I think some oncologists showing the recent evidence and references and high impact publications might help convince them to some degree that this is helpful.* Participant 4

Others noted that, despite the evidence supporting CGAs and having trainings available, there needed to be training opportunities that were accessible to more clinicians across various disciplines.

*I’ve completed online modules through SIOG and [institution] and just being part of CARG and as a personal research interest, but I agree…we do not really bring in the older adult geriatric type of thing in that specifically, ‘cause we have geriatric training in internal medicine, but it is like, 1 month in 3 years that it is required. And there is a geriatric fellowship, but that does not necessarily mean geriatric and cancer care area always overlapping for training purposes.* Participant 7

Participants acknowledged that uptake of CGA implementation can be impeded based on resistance from colleagues and that training for all cancer care clinicians and potential assessment implementers would help with this. Incorporating training into existing initiatives would encourage participation and implementation uptake.

*So there’s still a lot of work to be done to convince our colleagues because the pushback remains.* Participant 3

*I think that if it’s brought to a team like that and integrated into something we’re already going to be doing.* Participant 1

#### 3.2.5 Recommendations for training content and format

When answering the question, “What content would you value most in training/resources and preferred format?” participants mentioned videos, role-playing, and education. Participants also discussed the time constraints, highlighting the need to keep trainings short. Finally, participants also highlighted the ease of usability by incorporating it into existing educational infrastructures. Representative quotations were:

*Trying to keep it as concise as possible—for example, like a slide deck or something that you send out that people can go through at their convenience. But I think that if it was either somebody hired specifically for that role, then I think more of a short, intensive course would be really helpful.* Participant 8

*[I] think simple and easy to be learned, not time consuming. And visualization using video that’s perfect. And I would say also like focus more on the geriatric assessment.* Participant 6

*Workflows in EPIC that could save us time… [And] at our faculty meetings, just the awareness of hey, these tools are available.* Participant 1

Skills they would like to gain from trainings included better understanding how CGAs would specifically affect the patient. A participant mentioned that being more aware of the patient’s needs and how these outcomes will ultimately help patients and would be important to address in trainings.

*As far as the skills, what skills would I want to learn is, I do not know how to say this really but how having that piece of information is going to help this patient […] Just being aware of more of this patient’s experience.* Participant 1

Participants offered information about models at other institutions that could be beneficial for oncology providers to learn about.

*It is just having it happen at some of the key academic institutions that we all look to as being kind of thought leaders, and having them talk about the benefits that they’ve had. And I think, then, that everybody else will follow. I just think more experience and more attention to it is gonna be important.* Participant 8

## 4 Discussion

The purpose of this study was to explore the attitudes towards, experience with, and barriers and facilitators to CGA implementation amongst providers in oncology settings. Our findings revealed that providers view the CGA as an important tool to implement with cancer patients, particularly in assisting with treatment decisions and care planning. However, several important observations arose regarding the logistics of implementation, along with barriers and facilitators to its use in actual practice. The majority of participants indicated that the CGA took between 30–60 min to administer and ideally involved a multidisciplinary team (e.g., oncologist, physical therapist, occupational therapist, registered dietician, and/or advanced practice provider). Importantly, there was variability in when (before clinic visits, during intake visits, at follow-up visits), for whom (some implemented with select groups of patients), and how (some used select components or shorter screening questions rather than a full CGA) the CGAs were implemented. Notable barriers to implementation were lack of provider training/experience, time/resources, limited championing at diverse organizational levels, and limited incentives through billing. Conversely, key facilitators of implementation noted were having champions across multiple levels of the organization, provider education/training, multidisciplinary care teams, and institutional resources/buy-in. Lastly, notable preferences for CGA training for providers were a desire for tailoring content and the need for the training to be concise/low-burden.

Existing guidelines and recommendations agree on the utility of CGAs to identify potential toxicities and other adverse effects from treatment, life expectancy, non-oncologic health concerns, and psychosocial and supportive care needs for older adults with cancer (Mohile et al., 2018). What remains unclear is how CGAs can best be integrated into oncology clinic workflows and patient care. For example, while CGAs are recommended to identify several functional and psychosocial domains to be included, our participants reported wide variation in actual implementation. Some described the use of screening algorithms to identify patients who would benefit from a full CGA, and others discussed the targeted use of select CGA domains rather than the full assessment. This variation speaks to adaptation, often driven by the time, staff, and resource constraints oncology team members experience. The impact of these modifications in CGA implementation in relation to oncology care outcomes has yet to be evaluated.

Multidisciplinary team support is recognized as necessary to realize the full potential of CGAs to inform patient care (Presley et al., 2020). Yet, as our participants reported, such multidisciplinary involvement and collaboration does not always happen. Participants identified the lack of geriatric specialists as a key disciplinary gap for many oncology teams, one that will likely grow as the shortage of clinicians with geriatric expertise intensifies (Battisti and Dotan, 2020). It is also worth noting that most participants in this study were within academic medical centers or the VA and were potentially better resourced in terms of access to multidisciplinary expertise than many community hospitals where people receive cancer care. To address the need for multidisciplinary care that includes geriatric expertise, participants pointed to the need for education and training for non-geriatrics specialists. Organizations like the national Cancer and Aging Research Group have educational resources for use with clinical staff. In addition, geriatric oncology practices such as the multidisciplinary Cancer and Aging Resiliency Clinic (at The Ohio State University) that are structured around multidisciplinary care include geriatric-specific training for nurses (Presley et al., 2020); however, there are only 14 of such clinics in the United States, and most are in urban areas and near academic comprehensive cancer centers. Thus, accessing training targeted to oncology teams and having the time to do it remains challenging.

Institutional and administrative support is critical to addressing the barriers and challenges that participants reported. Such support will require evidence of the multi-level benefits of CGA use, including not just clinical outcomes, but other patient-reported benefits and institutional outcomes such as cost, such as hospital length of stay, ER use, and readmission rates. Yet, the evidence base for these outcomes in the context of cancer care is still relatively nascent and somewhat mixed.

One clear benefit that participants reported is patient involvement in their care, especially in discussions around treatment planning. Sustained, intentional survivorship care involvement for people with a cancer diagnosis is increasingly recognized as key to survivorship care planning and outcomes (Mead et al., 2020). For older cancer patients, the CGA could greatly facilitate patient-centered survivorship care planning.

### 4.1 Study limitations

Despite the study’s strength of providing a unique, in-depth exploration into providers’ perceptions and use of CGAs in cancer care, this study has limitations. The number of providers interviewed was small (eight interviews with nine participants), and only one participant represented primary care, limiting our ability to generalize findings more broadly. In addition, participants had a range of experience with implementing CGAs, and we did not assess the experience of support staff. However, the insights provided on the purpose and value of such assessments, along with the logistics and barriers to their use, are critically important for moving this field forward. Further, saturation was reached in the data regarding emergent themes, limiting the value of additional provider interviews. Despite study limitations, this in-depth qualitative data will help providers and healthcare systems consider what is needed to effectively implement CGAs with cancer patients and survivors.

### 4.2 Recommendations for research and practice

While there are inherent limitations with this relatively small study, it is still evident that the need for more research on the implementation of CGAs is warranted. The growing work in this area provides the foundation for recommendations to help advance the uptake of CGA use in primary and oncology care settings. Based on the literature, current guidelines, and this study’s findings, the following are potential strategies for future work on CGAs:

For researchers:

• In collaboration with clinical partners, complete an organizational readiness assessment (See example in Table 2) that will identify barriers and facilitators of organizational change needed to operationalize the routine use of CGAs as the standard of oncology care. Considerations for implementation include the use of a short screening tool to determine the need for a full CGA, administration of screening or full CGA prior to the visit if feasible (through EMR or at clinic intake), and whether support staff or the provider would administer the tool if not completed in advance (Friedman et al., 2020; Macauda et al., 2022).
• Evaluate modified and/or abbreviated CGA tools and methods to determine validity in terms of oncology care outcomes.
• Study the benefits to family caregivers of using CGA information to access services for their loved ones and themselves in managing cancer.
• Conduct a similar study in rural and smaller community health clinics.
• Re-analyze, through quantitative and qualitative research, the state of CGA practice in two to 3 years to identify best practices for successful implementation. This is especially important given the updated guidelines released following the completion of this study.

**TABLE 2:** Organizational readiness for geriatric assessment checklist for implementation. The following is a brief self-assessment of key factors that will be key to successful implementation of the geriatric assessment in your organization. Read each item and place a check mark to indicate whether or not you have it in place. If you do not presently have the factor in place, write down comments on the steps you will need to take.

Factors key to implementation	Yes	No	Comments
Staffing			
Leadership is supportive of the Geriatric Assessment			
Staff identified to deliver the Geriatric Assessment, with revisions to their position descriptions as needed			
Staff identified to supervise those completing the Geriatric Assessment			
Staff are designated to serve as the champion or implementation lead for the Geriatric Assessment			
An implementation team has been established for the Geriatric Assessment			
Staff training			
An outline of training objectives has been created for the Geriatric Assessment			
Staff have been identified to deliver the Geriatric Assessment training			
A training plan (time, location, curriculum, equipment) and tracking system has been created for the Geriatric Assessment training			
Recruitment			
A process and materials have been developed to recruit, refer, and otherwise reach individuals who would benefit from the Geriatric Assessment			
Partners engaged to help reach individuals who may benefit from the Geriatric Assessment			
Resources			
Space is available for conducting the Geriatric Assessment			
The space includes necessary equipment (chair, tape measure, handgrip dynamometer, etc.)			
A system is in place to document Geriatric Assessment processes and outcomes			
Any needed adaptations for the “reach” population have been identified and completed			
ASCO protocol and guidelines			
Communication			
Key stakeholders have been identified			
Communication plan across stakeholders is in place			
Monitoring and Evaluation			
Metrics have been identified for evaluation the Geriatric Assessment program			
An evaluation plan has been drafted specifying what data to be collected, when and by whom			

For clinicians and healthcare systems:

• Implement early and repeated CGAs as an integrated component of multidisciplinary oncology care. These efforts support oncology care that is tailored to the individual patient, facilitates patient-centered care with shared decision-making, and prioritizes optimization of quality and quantity of life as outcomes.
• Align CGA implementation efforts with the new 2023 ASCO Practical Assessment and Management of Vulnerabilities in Older Patients Receiving Systemic Cancer Therapy (Dale et al., 2023).
• Offer advanced training opportunities for practitioners on CGAs and effective implementation strategies for successful program delivery and impact. These include training both providers and support staff and assessment of available resources/actions based on CGA results to augment standard clinical decision-making.
• Relate CGA use to survivorship outcomes, such as hospital admissions/readmission, medication errors, or patient and caregiver quality of life to potentially influence policy and insurance billing practices for CGA implementation.

### 4.3 Conclusion

As the number of cancer survivors who are 65 and older continues to grow (Bluethmann et al., 2016), driven by the aging of the world’s population, earlier detection, more effective cancer treatments, and better supportive care, finding ways to reduce the human and global burden of cancer is becoming increasingly imperative. There has been a slow but steady shift in adult oncology away from a focus on life span or length of survival to one emphasizing health span, or the quality of the life to be lived. Introduction and uptake of processes like CGA to better identify those at risk of poor outcomes and intervening early to modify treatments and care to reduce preventable morbidity and mortality are important to consider for future research and practice if we are to maximize health span in the growing population of older cancer survivors living through and beyond their disease.

## Data Availability

The datasets presented in this article are not readily available because: Limited access to the data for this study (data collection instruments and codebook) is available. Requests to access the datasets should be directed to DBF, dbfriedman@sc.edu.
