# Supplementary material for: Examining provider perceptions and practices for comprehensive geriatric assessment among cancer survivors: a qualitative study with an implementation science focus
Source: Front Aging. 2023 Dec 4;4:1305922. doi: 10.3389/fragi.2023.1305922 (PMC10725930; doi:10.3389/fragi.2023.1305922)
Supplement: Supplementary file 1 [file DataSheet2.docx]

**Appendix 2: Interview Guide and Questions**

Thank you for your willingness to meet today. Since 2018, the gerontologic community, as well as the American Society of Clinical Oncology have recommended use of comprehensive geriatric assessment (CGAs) in the care of older adults with a history of cancer. However, there is great variability in the extent to which this is put into practice.

We are having discussions with providers to better understand your use of these assessments with cancer patients / survivors. The hope is that these discussions will help inform strategies, trainings, and the creation of resources for broader implementation of CGAs in primary care and oncology care settings, as well as to identify barriers to implementation.

To start, do you know if your care center performs CGAs? [Interviewer will circle YES or NO.]

If response is NO, Ask the following two questions:

1. Might you know why your care center is not currently conducting CGAs?
2. Can you share whether there have been discussions about whether to start implementing them with patients?

If response is YES, Begin the interview questions here:

1. How would you describe the purpose and value of a CGA?
2. What do you see as the benefits of performing a CGA with cancer survivors?
3. Do you describe the CGA to your patients? (Probe: All of them, including those with cognitive impairment?)
4. How do you describe the purpose of a CGA to patients? And how (if at all) is the description tailored by, for example, cognitive ability?
5. Who within your clinic is implementing CGAs?
6. When is a CGA administered? (Probes: Certain age? Health status? Triggered by acute event? Or regularly administered?)
7. Are all care center staff on board with implementation? (Probes: Is there someone specific championing this? Are leaders on board too? Is there resistance?)
8. Which specific assessment are you using within your clinic? Please name:_______ (Probes: review available EHR data? from organizations such as SIOG, NCCN, ASCO, etc.)
   1. Would you be willing to share a copy of the assessment form you are using?
   2. *If relevant, ask*: Is this a clinic-developed assessment/form and can you tell us how it was developed?
   3. How long does it take to implement the assessment you are using?
9. Do you know with what proportion (or percentage) of your clinic patient population is the CGA is being administered?
10. What categories or components of a CGA are typically implemented (ask YES/NO for each of the following)?
    1. Functional status check (ADLs/IADLs)
    2. Cognitive function assessment (MMSE? MoCA?)
    3. Geriatric Depression Scale
    4. Nutritional assessment (e.g., MNA)
    5. Gait and balance assessment (e.g., Timed Get up and go/TGUG)
    6. Cumulative Illness Rating Scale for Geriatrics
    7. Comorbidity Index (e.g., ACE 27)
    8. Qualify of life assessment (e.g., QLQ-C30)

[For any NO response in question 10 "a" to "h" ask question 11. If all are YES, then skip to question 12.]

1. In your opinion, what are the barriers to the components [specify from Question above] not being collected or implemented?
2. What is done with the information once it is collected? How do you document it? How is it analyzed and disseminated?
3. If a need is identified from the assessment, what are the next steps for your patients? (Probes: Refer patients to other services, Discuss how they impact the presenting issue, Change in care plan, etc.)
4. In your opinion, do you think all practitioners/medical settings should conduct CGAs, or are there specific practitioner types that should focus on this. Why do you think so?
5. What makes it difficult to implement CGAs?
6. What helps facilitate the implementation of CGAs?
7. What types of training (if any) have you received on implementing CGAs?
8. Do you feel that you would benefit from training/resources to help you and your clinic with implementing CGAs?

If so…

- 1. What content would you value most in training/resources?
  2. What skills would you want to gain from the trainings/resources?
  3. What type of format would you prefer for this?

1. What are some challenges you face when helping your patients and their families understand their CGA results?
2. Is there anything else that we may not have asked about or discussed that would help you with implementing CGAs with your cancer survivor patients?
3. Is there anyone else you recommend we speak with in either a primary care clinic or cancer care clinic for this initiative?

***Thank you for your time!***
